# Is it time to retire bolus cisplatin? Lessons from the ConCERT trial

**DOI:** 10.1093/jnci/djag331

**Published:** 2026-10-14

**Authors:** Ross D Merkin, Aarti Bhatia

**Affiliations:** Mass General Brigham Cancer Institute, Boston, MA, United States; Department of Medicine, Massachusetts General Hospital, Boston, MA, United States; Massachusetts Eye and Ear, Boston, MA, United States; Department of Medicine, Harvard Medical School, Boston, MA, United States; Department of Internal Medicine (Medical Oncology), Yale School of Medicine and Yale Cancer Center, New Haven, CT, United States

Definitive chemoradiation is the preferred curative treatment modality for head and neck squamous cell carcinomas of the oral cavity, oropharynx, hypopharynx, and larynx when surgery is technically challenging or can lead to functional impairment or organ loss. Cisplatin has been the standard radiosensitizer since the 1980s when the Radiation Therapy Oncology Group 81-17 trial demonstrated that concurrent chemoradiotherapy with cisplatin 100 mg/m^2^ every 3 weeks for 3 doses, colloquially referred to as “bolus” cisplatin, achieved a complete response rate of 71%.^1^ The preferred use of cisplatin over other radiosensitizing chemotherapy options is supported by the meta-analysis of chemotherapy in head and neck cancer (MACH-NC), which indicates that cisplatin monotherapy is associated with a similar magnitude of effect compared with doublet chemotherapy regimens. This meta-analysis was also among the first studies to note that lower cumulative cisplatin doses were associated with inferior outcomes.^2^ Concurrent chemoradiotherapy, although less morbid than surgical resection for some tumors, is nonetheless associated with substantial acute and late treatment toxicity.^3^ Improvements in radiotherapy (RT) technology, from 2D to 3D conformal radiotherapy, have measurably reduced radiation-related toxicity. For example, parotid-sparing intensity-modulated RT roughly halved the incidence of severe xerostomia compared with conventional RT in a randomized trial.^4^ Parallel efforts have sought to improve the tolerability of concurrent chemotherapy, primarily by modifying the dosing schedule of cisplatin, and a meta-analysis published in 2017 indicated that cisplatin administered at lower but more frequent doses was associated with similar efficacy and reduced toxicity.^5^ Low-dose weekly cisplatin was associated with significantly lower rates of nausea, vomiting, nephrotoxicity, and neutropenia. Collectively, given the observations of higher cumulative cisplatin dose being considered the primary driver of antitumor efficacy and weekly low-dose cisplatin being associated with improved tolerability, prospective head-to-head comparisons of bolus versus weekly cisplatin became justified.

The risk of on-treatment cisplatin toxicity is increased by peak concentration, explaining why a lower amount of cisplatin per dose, a slower infusion rate, and improved hydration status lower this risk.^6^^,^^7^ Weekly cisplatin is an appealing alternative to bolus, but whether lower toxicity would also be accompanied by inferior efficacy when prospectively compared head-to-head with bolus cisplatin was not known. Other prospective trials have randomly assigned patients to bolus versus weekly cisplatin. The Japan Clinical Oncology Group (JCOG) 1008 trial demonstrated noninferiority of weekly cisplatin 40 mg/m² to bolus cisplatin in the postoperative (adjuvant) setting.^8^ By contrast, Noronha et al.^9^ failed to demonstrate noninferiority of weekly cisplatin 30 mg/m² in a population receiving predominantly adjuvant therapy but that also included a smaller proportion of definitively treated patients, a finding most plausibly attributable to an inadequate cumulative dose at the lower weekly dosing schedule. Separately, additional trials have converged on 40 mg/m² as the preferred weekly dose without directly randomizing against bolus cisplatin: E3311 in the adjuvant treatment setting^10^ and NRG HN002^11^ and ORATOR2^12^ in the definitive treatment setting. These studies support weekly cisplatin at 40 mg/m² as an efficacious regimen but do not themselves establish noninferiority to bolus dosing. Until now, no completed trial had directly compared bolus and weekly cisplatin in the definitive setting; NRG-HN009 (NCT05050162) has a similar design but has not yet reported. It is in this context that the ConCERT trial^13^ was conceived.

ConCERT was a randomized, open-label, noninferiority phase 3 investigator-initiated trial of definitive concurrent chemoradiotherapy for the American Joint Committee on Cancer 8th edition stage III-IVB head and neck squamous cell carcinoma arising from the oral cavity, oropharynx, hypopharynx, and larynx. Patients were randomly assigned 1:1 to bolus cisplatin (100 mg/m² on days 1, 22, and 43) or weekly cisplatin (40 mg/m² weekly for up to 7 doses). Most patients received 2D cobalt-based RT, with a smaller proportion receiving 3D conformal RT with either intensity modulated RT or volumetric-modulated arc therapy; RT technique was balanced between arms. Chemotherapy toxicity was graded per CTCAE (Common Terminology Criteria for Adverse Events) v4.03 and RT toxicity per Radiation Therapy Oncology Group criteria. The primary endpoint was 2-year locoregional control; secondary endpoints included overall survival, progression-free survival (PFS), grade 3 or higher chemotherapy- and RT-related adverse events, and treatment adherence. The noninferiority margin was set at 10%. Across 6 sites in India, 278 patients were randomly assigned comprising a predominantly human papillomavirus (HPV)-independent population. The trial met its primary endpoint, demonstrating noninferiority of weekly cisplatin for 2-year locoregional control, and interestingly showing a similar signal to that seen in JCOG1008, with a trend toward improved locoregional control with weekly dosing (hazard ratio [HR] = 0.69, 95% confidence interval [CI] = 0.49 to 0.97; log-rank *P* = .035), while acknowledging the trial was not powered to test superiority; overall survival and PFS were similar between arms. ConCERT also demonstrated that weekly cisplatin was associated with statistically significantly less toxicity and better tolerability than bolus cisplatin: mucositis, myelosuppression, renal toxicity, vomiting, hyponatremia, and ototoxicity were either numerically or significantly more frequent in the bolus arm.

ConCERT was a well-conducted randomized phase 3 trial, and the authors deserve credit for completing it. Its results recapitulate the adjuvant-setting findings of JCOG1008 and represent the first completed phase 3 evidence of weekly cisplatin’s noninferiority specifically in the definitive setting. Notably, treatment interruptions and dose de-escalations were infrequent and occurred at similar rates in both arms. Chemotherapy completion rate, cumulative cisplatin dose received, and proportion of patients who received at least 200 mg/m² were numerically, but not significantly, higher in the weekly dosing arm. On the strength of its improved safety profile and noninferior efficacy, ConCERT supports the use of weekly cisplatin for definitive chemoradiation in HPV-independent head and neck squamous cell carcinoma, which predominated in this cohort. Because the study population was drawn from Indian centers with a low prevalence of HPV-associated oropharyngeal cancer, and did not include sinonasal or other rarer subsites, the trial cannot directly establish efficacy in those populations. Cisplatin 40 mg/m^2^ weekly nonetheless remains a standard radiosensitizer across head and neck subsites, and the broader weekly cisplatin literature, including E3311, NRG-HN002, and ORATOR2 in HPV-associated disease and JCOG1008 and Noronha et al.^9^ in mixed populations, makes it reasonable to extrapolate the ConCERT findings to the management of HPV-associated oropharyngeal cancer, pending direct confirmatory data. This trial meaningfully shifts the paradigm for how concurrent chemoradiation is administered.

We have learned from previous concerted investigative efforts that HPV-associated and HPV-independent head and neck squamous cell carcinomas have distinct tumor biology and disease course, and this context has led to divergent research hypotheses being evaluated based on HPV association. Given the superior prognosis of favorable-risk HPV-associated head and neck squamous cell carcinoma, recent trials in this subgroup of patients have aligned with an overarching goal of treatment de-escalation, for instance, de-intensified risk-adapted adjuvant therapy, as in E3311,^10^ and reduced-dose definitive RT, as in NRG-HN002.^11^ Efforts to substitute alternative systemic agents for cisplatin have uniformly demonstrated inferiority compared with standard cisplatin-based concurrent chemoradiotherapy, as exemplified in the RTOG 1016,^14^ De-ESCALaTE HPV,^15^ and the Trans Tasman Radiation Oncology Group (TROG) 12.01^16^ trials. Overall, current data support de-escalation of adjuvant therapy in HPV-associated oropharynx for selected patients but not definitive concurrent chemoradiotherapy.

Contrary to that, HPV-independent head and neck squamous cell carcinoma is associated with a worse prognosis, but despite endeavors at treatment intensification, only modest gains have been made in improving outcomes for these patients. Multiple trials explored the addition of systemic therapy agents to cisplatin and RT, including the epidermal growth factor receptor inhibitor cetuximab in RTOG 0522,^17^ lapatinib (epidermal growth factor receptor and HER2 inhibitor) in TRYHARD,^18^ anti–PD-1 inhibitors in the Javelin 100^19^ and Keynote 412^20^ trials, and the small-molecule inhibitor of apoptosis protein antagonist Xevinapant in the TrilynX trial.^21^ None of these agents added benefit to cisplatin and RT alone. Anti–PD-1 inhibitors, which have recently demonstrated improvement in event-free survival in the peri-operative treatment of stage III-IVA head and neck squamous cell carcinoma tumors with a PD-1 ligand combined positive score of at least 1,^22^^,^^23^ are now being investigated in the post concurrent chemoradiotherapy maintenance phase in patients with locally advanced HPV-independent and high-risk HPV-associated oropharynx cancer^24^ (NCT06129864, NCT03811015) to assess for improvement in event-free survival and PFS. Given the durable benefit seen with anti–PD-1 inhibitors, nivolumab was tested in combination with platinum-based chemotherapy in a single-arm proof-of-concept study in the induction setting in patients with HPV-associated oropharynx cancer.^25^ Among 73 eligible patients, 70.8% had deep responses, allowing for treatment de-intensification with single modality RT or transoral robotic surgery or intermediate-dose concurrent chemoradiotherapy to 45-50 Gray. This may be a strategy that merits exploration in a late-phase trial. While the quest for more effective and better tolerated therapeutic approaches remains active, ConCERT offers proof that weekly cisplatin can maintain efficacy and improve tolerability of an aggressive but necessary treatment.

In summary, ConCERT is the first completed randomized phase 3 trial to prospectively compare bolus (100 mg/m²) with weekly (40 mg/m²) cisplatin in the definitive chemoradiation setting. It supports weekly cisplatin as an evidence-based, better-tolerated alternative to the traditional bolus regimen in HPV-independent disease, with reasonable though not yet directly proven extrapolation to HPV-associated disease. This is a practice-changing study. Future work should focus on optimizing usage of established biomarkers like PD-1 ligand combined positive score and HPV status, along with emerging tumor multi-omics and ultrasensitive circulating tumor DNA assays as strategies that could further tailor therapy by disease risk and select patients for treatment de-escalation or intensification.

## Data Availability

No new data were generated or analyzed for this editorial.
